# Reporting Diagnostic Reference Levels for Paediatric Patients Undergoing Brain Computed Tomography

**DOI:** 10.3390/tomography9060159

**Published:** 2023-11-01

**Authors:** Ali Alhailiy, Essam Alkhybari, Sultan Alghamdi, Nada Fisal, Sultan Aldosari, Salman Albeshan

**Affiliations:** 1 Department of Radiology and Medical Imaging, College of Applied Medical Sciences, Prince Sattam Bin Abdulaziz University, Alkharj 11942, Saudi Arabia; e.alkhybari@psau.edu.sa; 2 Radiology and Nuclear Medicine Department, Security Force Hospital, P.O. Box 3643, Riyadh 11481, Saudi Arabia; sultan-661@hotmail.com; 3 Radiology Department, King Fahad Medical City, P.O. Box 59042, Riyadh 11525, Saudi Arabia; duramatter_999@hotmail.com; 4 Medical Imaging Department, King Saud Medical City, Riyadh 12746, Saudi Arabia; su.aldosari@ksmc.med.sa; 5 Radiological Sciences Department, College of Applied Medical Sciences, King Saud University, P.O. Box 145111, Riyadh 4545, Saudi Arabia; salbeshan@ksu.edu.sa

**Keywords:** computed tomography, diagnostic reference levels, paediatric CT imaging, weight groups

## Abstract

Brain computed tomography (CT) is a diagnostic imaging tool routinely used to assess all paediatric neurologic disorders and other head injuries. Despite the continuous development of paediatric CT imaging, radiation exposure remains a concern. Using diagnostic reference levels (DRLs) helps to manage the radiation dose delivered to patients, allowing one to identify an unusually high dose. In this paper, we propose DRLs for paediatric brain CT examinations in Saudi clinical practices and compare the findings with those of other reported DRL studies. Data including patient and scanning protocols were collected retrospectively from three medical cities for a total of 225 paediatric patients. DRLs were derived for four different age groupings. The resulting DRL values for the dose–length product (DLP) for the age groups of newborns (0–1 year), 1-y-old (1–5 years), 5-y-old (5–10 years) and 10-y-old (10–15 years) were 404 mGy cm, 560 mGy cm, 548 mGy cm, and 742 mGy cm, respectively. The DRLs for paediatric brain CT imaging are comparable to or slightly lower than other DRLs due to the current use of dose optimisation strategies. This study emphasises the need for an international standardisation for the use of weight group categories in DRL establishment for paediatric care in order to provide a more comparable measurement of dose quantities across different hospitals globally.

## 1. Introduction

Computed tomography (CT) is a vital diagnostic imaging modality utilized in radiology departments to investigate numerous pathological conditions by displaying anatomical cross-sectional CT images [1]. CT procedures commonly involve the delivery of higher radiation doses than X-ray radiographic procedures. A wide range of published articles acknowledge that paediatric CT procedures might potentially induce cancer risk [2,3]. In the last twenty years, numerous international organizations have supported the optimization of radiation doses delivered for diagnostic purposes in medical procedures, particularly CT procedures. These organizations include the European Basic Safety Standards, the International Atomic Energy Agency (IAEA), the International Basic Safety Standards (BSS), and the International Commission on Radiological Protection (ICRP). The primary goal of these standards is to minimize the radiation risks associated with the radiation burden while obtaining a suitable diagnostic image quality in diagnostic medical examinations. Furthermore, the monitoring of CT radiation exposure is essential, particularly in paediatric CT imaging. This is because the paediatric population is more vulnerable to radiation, particularly at a younger age [4]. Therefore, the optimization of CT radiation doses for children is very important for lowering radiation doses, as well as the radiation risk, while obtaining an optimal diagnostic image quality [5,6].

The diagnostic reference level (DRL) method is applicable to CT diagnostic procedures, and it has been promoted as a practical clinical tool for use in the optimization process of radiation dose delivery from diagnostic equipment [7]. DRLs have been widely employed to develop appropriate radiation safety protection measures for paediatric patients undergoing CT procedures, X-ray procedures, interventions, and nuclear medicine procedures. On the national level, many countries have applied the DRL concept using the basic strategy of performing national radiation dose surveys for different paediatric CT procedure types, and they have established national DRLs (NDRLs) recommended by international organizations, such as the International Commission on Radiation Protection (ICRP Publication 135) and European Guidelines on Diagnostic Reference Levels for Paediatric Imaging. These organizations also encourage the use of NDRLs based on weight and age group categories or the use of the age paediatric category for the purpose of previous data comparison with the existing, published NDRLs [5,6].

Two dosimetric radiation dose quantities are recommended in ICRP Publication 135 and the EG guidelines for the establishing of DRLs. These are the volumetric CT dose index (CTDI_vol_) and dose–length product (DLP). The CTDI_vol_ measures the radiation dose delivered to a patient during a CT procedure. The CTDI_vol_ unit reflects both the radiation intensity and the scan range, and it is expressed in milligray (mGy) units. In clinical practice, the CTDI_vol_ radiation dose metric value is commonly displayed on the CT monitor. The DLP radiation dose metric is a measure of the total radiation burden delivered to the patient throughout the entire CT procedure, and the DLP unit is mGy cm. The DLP mathematical formula consists of multiplying the CTDI_vol_ radiation dose matric by the length of the CT procedure. It enables an evaluation of the total radiation burden delivered to the patient undergoing the CT scan [5].

The DRL is determined at the 75th percentile of the CT radiation dose quantity used in CT paediatric imaging procedures during surveys [5,6]. For the investigation of the radiation doses, each paediatric CT imaging department participating in the NDRL survey should compare their median radiation dose quantity against the NDRLs. When the median radiation dose quantity exceeds the derived NDRL standard, the paediatric CT imaging centre should employ all applicable measures to optimize the CT radiation dose by changing the department paediatric protocol or upgrading their CT scanners [6,7,8].

In Saudi Arabia, the Saudi Food and Drug Authority (SFDA) has only established NDRLs for CT examinations in the adult population [9]. A few DRL studies for paediatric CT scans have been published in Saudi Arabia, focusing on the abdomen, chest, and pelvis, with limited publications aiming to evaluate DRLs for paediatrics in CT brain imaging [10,11]. It is crucial to evaluate whether the paediatric CT brain scans currently being performed align with practices in other areas of the country and internationally. Thus, in this study, we aimed to derive DRLs for CT brain procedures performed in paediatric centres.

## 2. Materials and Methods

Ethical approval was granted by the institutional review boards (IRBs) of the individual hospitals recruited for this study. Out of four medical cities, three hospitals completed and returned the survey as an Excel sheet. Five types of MDCT (multi-detector computed tomography) systems provided by three different CT scanner manufacturers were included: the 64-slice and 256-slice revolution systems of General Electric, the Siemens and Philips iCT system, and the 128-slice Siemens Definition Flash system.

### 2.1. Data Collection

The data collection survey served to collect standard information related to brain CT scanning parameters and the demographic information of the paediatric populations from each hospital, including data related to the tube potential (kVp), tube current (mA), rotation time, pitch, slice thickness, scan length, and level of iterative reconstruction (IR) for each patient. Patient characteristics (namely, age, gender, weight, height, and cross-sectional area (CSA) measurements) were also recorded if available. The DLP and CTDI_vol_ were recorded from the dose report as the two main dose descriptors recommended for reporting DRLs.

The datasets were collected retrospectively from the Radiology Information System (RIS) and Picture Archiving and Communication System (PACS). These included data on recent brain CTs performed for paediatric patients (newborn to 15 years of age). Indications included all CT examinations without contrast enhancement for the evaluation of seizures, trauma, headache, hydrocephalus, and pre- and post-PV shunt and to assess the spaces occupied by lesions. CT angiogram procedures for the evolution of the Circle of Willis were excluded, as they require a higher dose than that used for a standard helical brain CT, which would have skewed the results. A previous review noted that there is a lack of standardization of age group categories in the protocols for reporting for paediatric DRLs [1]. Thus, in the current study, we established paediatric DRLs based on the age groups recommended in the ICRP Publication 135 guidelines [5]. The age groups were 0, 1, 5, 10, and 15 years. Data from 226 paediatric patients were used in the analysis, as follows: <1 year (n = 60), >1- to < 5 years (n = 59), >5- to <10 years (n = 60), and >10–15 years (n = 47).

### 2.2. Statistical Analysis

The datasets were analysed using the Statistical Package for the Social Sciences (SPSS) software (v22.0, IBM Corp. Armonk, NY, USA). The dose-length product (DLP), body size descriptors, and weight and CSA measurements were tested for normality using histogram visual assessments and plots. Hence, an independent-sample *t*-test was used to compare the DLP and body size parameters between hospitals. The analysis showed no significant variation in these factors. The *p*-values for the weight and CSA measurements were 0.5 and 0.22, respectively.

The median CTDI_vol_ and DLP for each age group were calculated, and the DRL value was reported as the 75th percentile. Additionally, both the 50th and 25th percentiles were also reported as the most common indicators representing DRLs [12]. Despite the fact that the tube current has a strong effect on dose quantities, the effect of the tube current was not assessed in this study, as different CT scanners were used; therefore, different Focus Axis Distances (FADs) were applied, which would have skewed the tube current values. The relationship between the dose indicator (DLP), weight, and CSA for the sample was assessed using Pearson correlation analysis.

## 3. Results

A total of three hospitals participated in this study, and datasets comprising 226 paediatric patients were used in the analysis. In total, 61% of patients were boys and 39% were girls, with an average age of 8 years for both genders. In addition, the population’s weight and CSA were normally distributed among the paediatric age groups. A noticeable variation in CSA was noted between the scanning protocols applied for different age groups, with a median value of 169 mm and range of 100–290 mm. The median values of the CTDI_vol_ and DLP for the CT examinations increased consistently from smaller to larger patient sizes. The most frequent indications for head CT imaging were trauma (24%), hydrocephalus on VP shunt (23%), follow-up post-operation (13%), cerebral insult and seizure (11.5%), paediatric movement disorder (7%), craniosynostosis and macrocephaly (6%) and abscesses or tumours (6%). Headache and other indications were reported for 9.5% of the paediatric patients.

There were significant variations in the exposure factors applied within and across the hospitals. The tube current (mA) varied for all age groups: 34–349 mA (<1 year); 33–369 mA (1 year); 19–349 mA (5 years); and 33–474 mA (10 years). A tube voltage range from 100 to 140 kVp was used for all paediatric age groupings except for 5% of patients in the age group of 5 years, for which we used a lower value of 80 kVp. The characteristics of the paediatric patients and scanning features are shown in Table 1 and Table 2.

The Pearson’s correlation analysis showed a strong positive association between weight and CSA (r = 0.63, *p* = 0.001), with a coefficient of determination of 72%. Furthermore, a moderate positive correlation of the DLP with both weight and CSA was noted (r = 0.20 and 0.34, respectively, *p* = 0.001).

**Figure 1:**
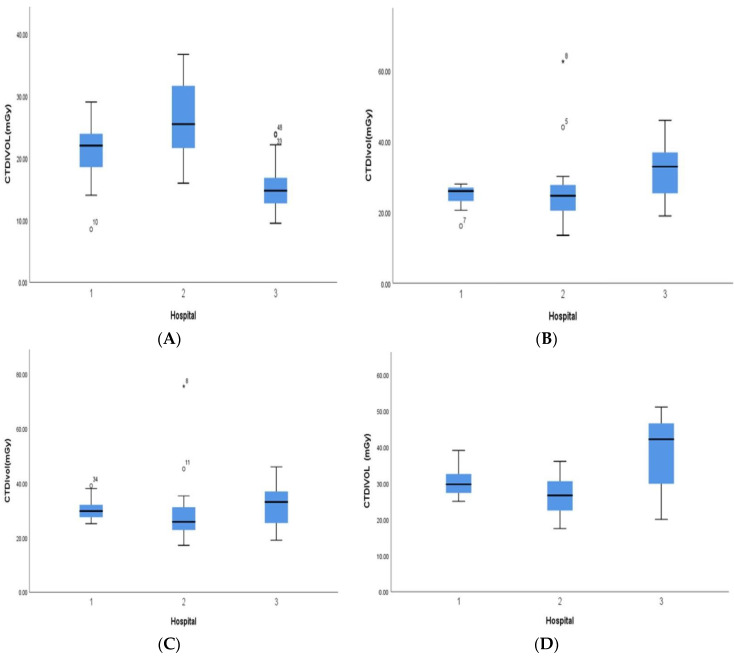
Distribution of volume computed tomography dose index (CTDI_vol_) per centre for age group of <1 year (**A**), 1 years (**B**), 5 years (**C**) and 10 years (**D**).

**Figure 2:**
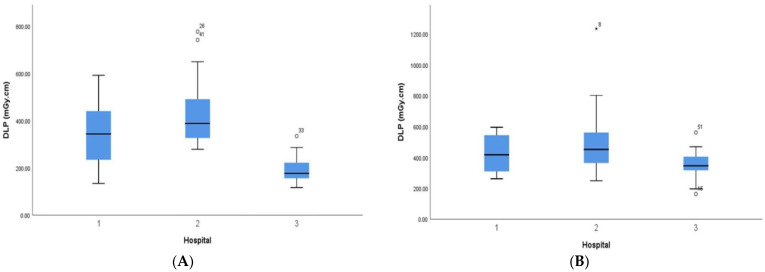

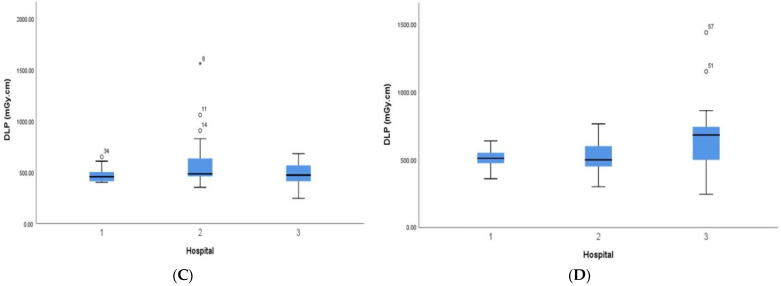
Distribution of dose–length product (DLP_l_) per centre for age group of <1 year (**A**), 1 years (**B**), 5 years (**C**) and 10 years (**D**).

**Figure 3:**
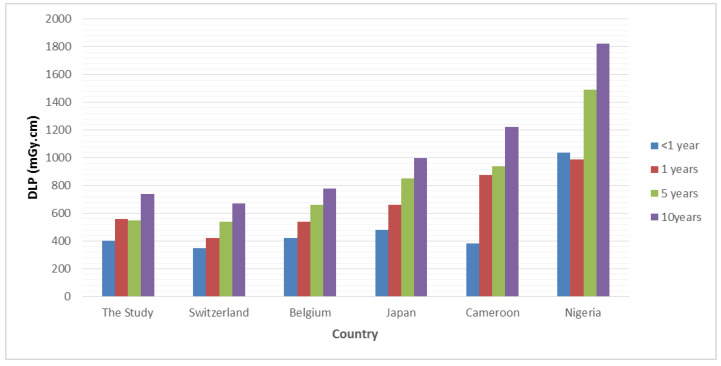
DRL values in DLP from this study compared with other NDRLs including Switzerland [13]; Belgium [14]; Japan [15]; Cameroon [16] and Nigeria [17].

The dose variations across the hospitals for each of the age groupings and their related DRL values (75th percentile) are presented in Figure 1 and Figure 2. The radiation dose value ranges for all age groupings were as follows: CTDI_vol_: 9–37 mGy, DLP: 117–776 mGy cm (<1 year); CTDI_vol_: 14–63 mGy, DLP: 245–1233 mGy cm (1 year); CTDI_vol_: 17–75 mGy, DLP: 245–1560 mGy cm (5 years); and CTDI_vol_: 18–51 mGy, DLP: 245–1436 mGy cm (10 years). The resulting values of the 75th percentile, median, and 25th percentile for both the CTDI_vol_ and DLP are summarised in Table 3. A comparison of the results with the NDRLs reported from other countries is shown in Figure 3.

## 4. Discussion

The current recommendation from the International Commission of Radiation Protection (ICRP) introduced DRLs as a tool with which to validate dose optimization strategies so as to avoid unnecessary patient radiation exposure while maintaining an acceptable image quality [8]. Furthermore, ICRP Publication 135 and the European paediatric DRL project recommend age and weight groups for establishing DRLs, as these indicators are used widely in CT protocol optimization in paediatric imaging [5,6]. However, several reviews have shown a variation in age-based groupings in establishing DRLs for paediatric brain CT examinations. Few NDRL studies followed age group ranges, such as <1 year, 1–5 years, 6–10 years, and 11–15 years, while, in other studies, grouping ranges of <5 years, 5–10 years, and more than 10 years were applied [1]. In this study, the age group range was in line with that recommended in ICRP Publication 135 for establishing DRLs for accurate local and international comparisons of dose quantities [2,18].

This study also indicated significant dose variations among the included hospitals for similar age groupings. Despite the fact that there were some hospitals with a narrow range of dose quantities, most paediatric age groups showed inconsistencies in their CTDI_vol_ and DLP values within and between hospitals (Figure 1 and Figure 2); the highest was a twofold difference in the median DLP between hospitals (177–388 mGy cm) in the age group of patients under one year. This can be explained by differences in the mAs and kVp used in the scanning techniques across the hospitals and differences in head size determined by the CSA values for the same age group. Among those hospitals surveyed, the lowest median DLP was noted for hospital 3, with 177 mGy cm and 346 mGy cm for the <1 year and 1 year age groups, respectively. A possible explanation for this low DLP is the fact that more than 45% of the brain CTs were performed with a kVp value of ≤100 and mAs 10% lower than the mean mAs for all scans in each age group.

Several scanning parameters had tremendous effects on the dose quintiles for the paediatric CT examinations, including exposure factors, slice thickness, pitch value, the use of ATC modulation, the type and level of iterative reconstruction, and, most importantly, the scan range. Despite the variation in the kVp values and scan range within and between hospitals, our analysis showed consistencies in other scanning parameters and applied dose-saving techniques.

It is important to note that the scan length must be minimized according to the clinical symptoms to be investigated, as it considerably affects the doses delivered to patients during CT exams [19]. The Z-axis coverage for a routine brain CT examination is usually from the apex to the base of the skull. Our literature search showed a lack of information on scan length for brain CTs based on recent NDRL studies. One study’s authors argued that the typical scan length for brain CT in patients aged 10 years is less than 14 cm [17]. However, Ekpo et al. suggested that the scan length determined from the collected DLP and CTDI_vol_ data for this age group may reach 29 cm on average. In this study, the average scan length for brain CT among children aged 10 years was 18 cm. Thus, it is logical to conclude that the DRLs in our study were significantly lower than those in the previous study [17], accounting for the 43% and 92% variations in the CTDI_vol_ and DLP, respectively, for the selected age group of 10 years.

Weight grouping criteria can be used as an alternative option for establishing paediatric DRL surveys if age information cannot be obtained [6]. However, ICRP Publication 135 outlines that weight in the paediatric population can vary by a factor of 100 from a newborn baby to a large adolescent, which makes the use of weight bands in DRL establishment challenging [5]. This broad range of paediatric weights emphasises the need for evaluations to determine which body size descriptors significantly impact patient dosage. The results from this assessment can contribute significantly to efforts to define groups of children for the purpose of paediatric dose surveys and the establishment of DRLs.

Although several studies have observed corrections of CT dose with patient weight and body mass index (BMI) [20,21], it does appear they are not ideal body size indicators for grouping paediatric patients, as the head cross-sectional diameter changes less with age than with weight and BMI [5]. To overcome this issue, several researchers have suggested that using body size measurements, such as the anteroposterior and transverse dimeters of the head, is preferable to their use in paediatric patient grouping.

For the first time, the current work provides CSA measurements for paediatric brain CT. The results of the regression analysis in our study indicate that head size measurements, such as CSA, predicted the DLP significantly better than weight indicators, which had a less significant influence on the DLP. Furthermore, several studies have indicated that CSA is a stronger predictor of the DLP, whilst patient weight and age have less effect on radiation dose quantities [22,23]. It is important to note that during the data collection period, the participating radiographers had to spend a lot of time gathering weight and height information from the patients’ electronic records, because these parameters were not included in the PACS system. This can considerably diminish the amount of data available and extend the time required to collect data for a DRL survey. Additionally, a previous study conducted by the NDRLs in Australia found that radiation dose quantities varied significantly in hospitals that did not have weight restriction criteria due to the wide range of patient sizes examined [24].

CSA can be considered an alternative measure of patient size and age for setting DRLs and dose optimisation strategies in CT examinations, as these studies involve significant differences in physical body habitus [23]. For head CT imaging, in particular, the concept of using CSA measurements is crucial, because head circumference measurements vary widely in many ethnic groups [25]. Measurements of head circumference can be obtained using the digital callipers on the CT display console from the midventricular axial CT slice, which displays the most prominent frontal horns of the lateral ventricle. This selected CT slice is correctly used for the estimation of the cranial CSA, as it reflects the maximum head circumference [26].

Larger DRL studies and dose surveys across the world have established DRLs for paediatric brain CT imaging and demonstrated wide variation in DRL values, as well as variations in the methods used for establishing DRLs, particularly in weight/age-based grouping [1]. This uniformity in weight and age grouping makes it difficult to compare DRL results with other NDRLs globally. Compared to other recently published DRL studies that followed a similar age grouping to that recommended in ICRP Publication 135, relatively low values were observed in this study (Figure 3). The doses are 40% lower than the data published in Nigeria [17], Japan [15], and Cameroon [16] but comparable to those from Switzerland [13] and Belgium [14]. This low value can be attributed to the uniformity of the head scanning protocols in which the current dose-saving technologies are applied. For example, IR and ATC modulation was used for 100% of patients in a routine scanning protocol. The results of that study are in line with several DRL studies conducted in Saudi hospitals, suggesting a good awareness of the principles of radiation protection in medical imaging practices in Saudi Arabia [27].

A limitation of this work is that a small number of hospitals were surveyed. This is because the Saudi Food and Drug Authority (SFDA) is currently collecting data on the most common types of CT examinations to establish national dose surveys for both adult and paediatric patients [9]. However, the SFDA has not yet published any DRL CT data for paediatric patients. Our proposed dose survey provides recommendations for radiation safety authorities and related bodies that may be used to conduct national dose surveys for paediatric brain CT examinations in Saudi hospitals.

In light of the rapid improvements in CT imaging technology, we recommend that the SFDA and hospitals continuously update the DRLs for paediatric CT examinations for at least the next 3 years in order to monitor radiation exposure and the potential risk of radiation-induced diseases from CT in paediatric patients in Saudi Arabia.

## 5. Conclusions

The data show that the DRL values described here are comparable to or slightly lower than those in European countries and Japan. There was a noticeable variability in the facility DRLs between the hospitals surveyed, which can be attributed to the current dose-saving methods used in scanning protocols, such as those using ATC modulation, IR, and a low kVp. Our findings suggest that size-specific dose estimate (SSDE) values should be considered as dose indicators and proposed in any future DRL dose surveys in order to provide a clinically important source of information for the optimisation of paediatric brain CT examinations. The outcome of this study provides baseline guidance information for establishing NDRLs for paediatric brain examinations in Saudi Arabia.

## Figures and Tables

**Table 1:** Patient characteristics (n = 226).

Patient Characteristics		ICRP Age (Years) Mean (% or Interquartile Range)			
		<1	1	5	10
No. of patients		60	59	60	47
Patient age, years		6 M (IQR 2.9)	3 Y (2.4)	7 Y (6.9)	13 Y (11.15)
Gender	Boy	33 (55%)	31 (52.5%)	44 (73%)	30 (64%)
	Girl	27 (45%)	28 (47.5%)	16 (27%)	17 (36%)
Patient height, cm		61.9 (IQR 53.5–69.3)	95.5 (IQR 85.5–108)	123.7 (IQR 115–135)	135.1 (IQR 134.3–146.8)
Patient weight, kg		6.4 (IQR 3.5–8.1)	14 (IQR 11–16.6)	25.2 (IQR 19.8–27.2)	44.7 (IQR 36.5–55)
Body mass index, kg/m^2^		15.7 (IQR 13–17.4)	15.2 (IQR 13.9–16.5)	17.6 (IQR 14.4–19.4)	20.5 (IQR 16.3–21.6)
Transverse width, mm		111.3 (IQR 92.4–124.5)	132.3 (IQR 126–137.7)	139.2 (IQR 132.1–144.7)	141.6 (IQR 134.7–148.3)
AP width, mm		130.1 (IQR 114–140.6)	156 (IQR 148–167.6)	168.4 (IQR 160.9–177.1)	170.7 (IQR 165.4–176.5)
Cross-Sectional Area, cm^2^		115.5 (IQR 83.5–142.1)	163.4 (IQR 146.4–183.1)	183.8 (IQR 174.4–191.9)	190 (IQR 180–200.6)
scan length, mm		154.7 (IQR 121.3–190)	177.5 (IQR 142.6–210)	179.4 (IQR 143.2–210)	191.8 (IQR 162.7–220)

AP width, anterior posterior diameter; IQR, Interquartile Range.

**Table 2:** CT scanning protocol and dose characteristics.

Scanning Characteristics	ICRP Age (Years) Mean (% or Interquartile Range)			
	<1	1	5	10
Tube voltage, kV	100 Median (IQR 100–120)	120 Median (IQR 100–120)	120 Median (IQR 100–120)	120 Median (IQR 120–120)
Tube current, mA	176.6 (IQR 125–243.5)	183.5 (IQR 125–247)	182.6 (IQR 145–249)	173.8 (IQR 140–210)
slice thickness	2.5 Median (IQR 2.5 –5)	2.5 Median (IQR 2.5 –5)	2.5 Median (IQR 2.5–5)	2.5 Median (IQR 2.5–5)
Pitch	0.53 Median (IQR 0.5–1)	0.65 Median (IQR 0.5–1)	0.65 Median (IQR 0.5–1)	0.65 Median (IQR 0.5–1)
IR	60 (100)	59 (100)	60 (100)	47 (100)
CTDI_vol_	20.9 (IQR 15.1–24.5)	27.6 (IQR 22.7–30.7)	30.5 (IQR 25.2–34.5)	32.3(IQR 27–38.1)
Total DLP, mGy cm	312.6 (IQR 189.3–403.9)	470.3 (IQR 365–561.4)	519.5 (IQR 418.8–554.9)	574 (IQR 454–681)

DLP, dose–length product.

**Table 3:** DRLs for Brain CT for pediatric for different age grouping.

Age Group	CTDI_vol_ (mGy)			DLP (mGy cm)		
	75th	Median	25th	75th	Median	25th
<1 year	25	22	15	404	281	191
1 years	30	26	23	560	452	367
5 years	34	29	25	548	474	426
10 years	45	30	20	742	551	439

CTDI_vol_, volume CT dose index; DLP, dose–length product.

## Data Availability

A full dataset is available upon request to the corresponding author.
